# Supplementary material for: Individuals with fibromyalgia have a different gait pattern and a reduced walk functional capacity: a systematic review with meta-analysis
Source: PeerJ. 2022 Mar 21;10:e12908. doi: 10.7717/peerj.12908 (PMC8944336; doi:10.7717/peerj.12908)
Supplement: Supplemental Information 2 [file peerj-10-12908-s002.docx]

**Systematic Review and/or Meta-Analysis Rationale**

*The rationale for conducting the systematic review / meta-analysis.*

Fibromyalgia syndrome (FM) is a rheumatic disorder that affects 3-5% of the population. FM is associated with altered sensory responses and previous studies have shown deficits in functional abilities.

The gait is a test that is routinely used to measure functional capacity. There are many standardized tests that are used to measure gait as well as different instruments that allow parameterization of gait. No systematic review or meta-analysis has been carried out that qualitatively and quantitatively has analyzed how FM affects walking.

*The contribution that it makes to knowledge in light of previously published related reports, including other meta-analyses and systematic reviews.*

Having analyzed 31 studies, for a cumulative total of 4078 participants, identifying significant differences greater than 20% compared to healthy subjects. Furthermore, qualitatively alterations have been identified that could justify the lower functional efficiency in people suffering from FM.
